# Impact of a combined case-based and evidence-based medicine teaching approach in traditional Chinese orthopedic residency training: a single-center, retrospective educational study

**DOI:** 10.3389/fmed.2026.1780896

**Published:** 2026-03-30

**Authors:** Yun Zhang, Yu Zeng

**Affiliations:** 1 Department of Traditional Chinese Medicine, Sichuan Provincial People’s Hospital, University of Electronic Science and Technology of China, Chengdu, China; 2 Department of Hyperbaric Oxygen, Sichuan Provincial People’s Hospital, University of Electronic Science and Technology of China, Chengdu, China

**Keywords:** case-based teaching, evidence-based medicine, orthopedics, retrospective educational study, traditional Chinese medicine

## Abstract

**Background:**

This single-center, retrospective study explored the effectiveness of case-based teaching combined with evidence-based medicine-based teaching in traditional Chinese orthopedic residency training to provide a theoretical basis for developing clinical training programs in traditional Chinese orthopedics.

**Methods:**

This retrospective study included 102 orthopedic residents undergoing residency training in traditional Chinese orthopedics at our hospital from January 2022 to December 2024. All data were obtained from the hospital management information system and were divided into a traditional group (*n* = 50, traditional teaching method) and a combined group (*n* = 52, case-based teaching combined with evidence-based medicine-based teaching). The study compared the theoretical and practical examination scores, critical thinking abilities, medical students’ self-learning abilities, and teaching quality between the two groups.

**Results:**

The combined group outperformed the traditional group in both theoretical and practical examination scores. After the training, the combined group scored higher than the traditional group in critical thinking ability assessments related to analytical thinking, systematic thinking, open-mindedness, curiosity, self-confidence, pursuit of truth, and cognitive maturity, as well as in overall scores. The self-practice ability scores in self-expression ability, self-learning ability, self-cultivated interest, teamwork ability, innovative adaptability, and clinical diagnostic and therapeutic ability were higher than those of the traditional group. Additionally, the teaching quality scores and total scores in learning interest, learning ability, learning attitude, learning thinking, and operational proficiency were also higher than those of the traditional group, with statistically significant differences (*P* < 0.05).

**Conclusion:**

The combination of case-based teaching and evidence-based medicine-based teaching in traditional Chinese medicine orthopedic residency training enhances teaching effectiveness, improves students’ critical thinking skills, self-learning abilities, and teaching quality.

## 1 Introduction

Residency training is pivotal in medical education, bridging theoretical knowledge with clinical practice to cultivate competent physicians (1). Traditional Chinese Medicine (TCM) orthopedics, characterized by intricate theories, specialized techniques, and a strong clinical focus, presents unique teaching challenges (2). Conventional lecture-based teaching often struggles to address the complexity of TCM orthopedic knowledge and may not adequately foster the clinical reasoning skills required for modern practice (3). As the aging population increases the burden of musculoskeletal disorders, there is a pressing need to enhance the clinical preparedness and critical thinking abilities of TCM orthopedic residents (4).

To address these pedagogical gaps, educators have explored various teaching innovations. Case-based teaching (CBT) grounds learning in realistic clinical scenarios, promoting active engagement and problem-solving skills (5). Evidence-based medicine (EBM) training emphasizes the critical appraisal and application of the best available research evidence to inform clinical decisions (6). Integrating CBT with EBM principles offers a promising, synergistic approach. CBT provides the authentic context for applying clinical questions, while EBM provides the methodological framework for seeking and evaluating evidence to answer them. This combination has shown promise in fostering deeper understanding and better clinical reasoning in other medical disciplines (7).

However, the application and evaluation of such an integrated CBT-EBM teaching model specifically in TCM orthopedic residency training remain largely unexplored. Most existing studies focus on either CBT or EBM in isolation, or within broader medical or Western orthopedic contexts. There is a scarcity of empirical evidence regarding the combined approach’s effectiveness on key educational outcomes in the specialized domain of TCM orthopedics (8–11).

Therefore, this single-center, retrospective study aimed to evaluate the impact of a combined CBT and EBM teaching approach on the theoretical knowledge, practical skills, critical thinking ability, self-directed learning capacity, and perceived teaching quality of TCM orthopedic residents, compared to a traditional lecture-based teaching model.

## 2 Materials and methods

### 2.1 Ethical statement

This retrospective educational study was conducted in accordance with the Declaration of Helsinki and was approved by the Ethics Committee of Sichuan Provincial People’s Hospital (Approval No. 2024091010). The requirement for informed consent was formally waived by the ethics committee due to the retrospective nature of the study. All data used were anonymized educational assessment data retrieved from the hospital’s management information system, and the analysis involved no intervention or risk to the participants. All data were handled confidentially and used solely for the purpose of this research.

### 2.2 Study design

**FIGURE 1:**
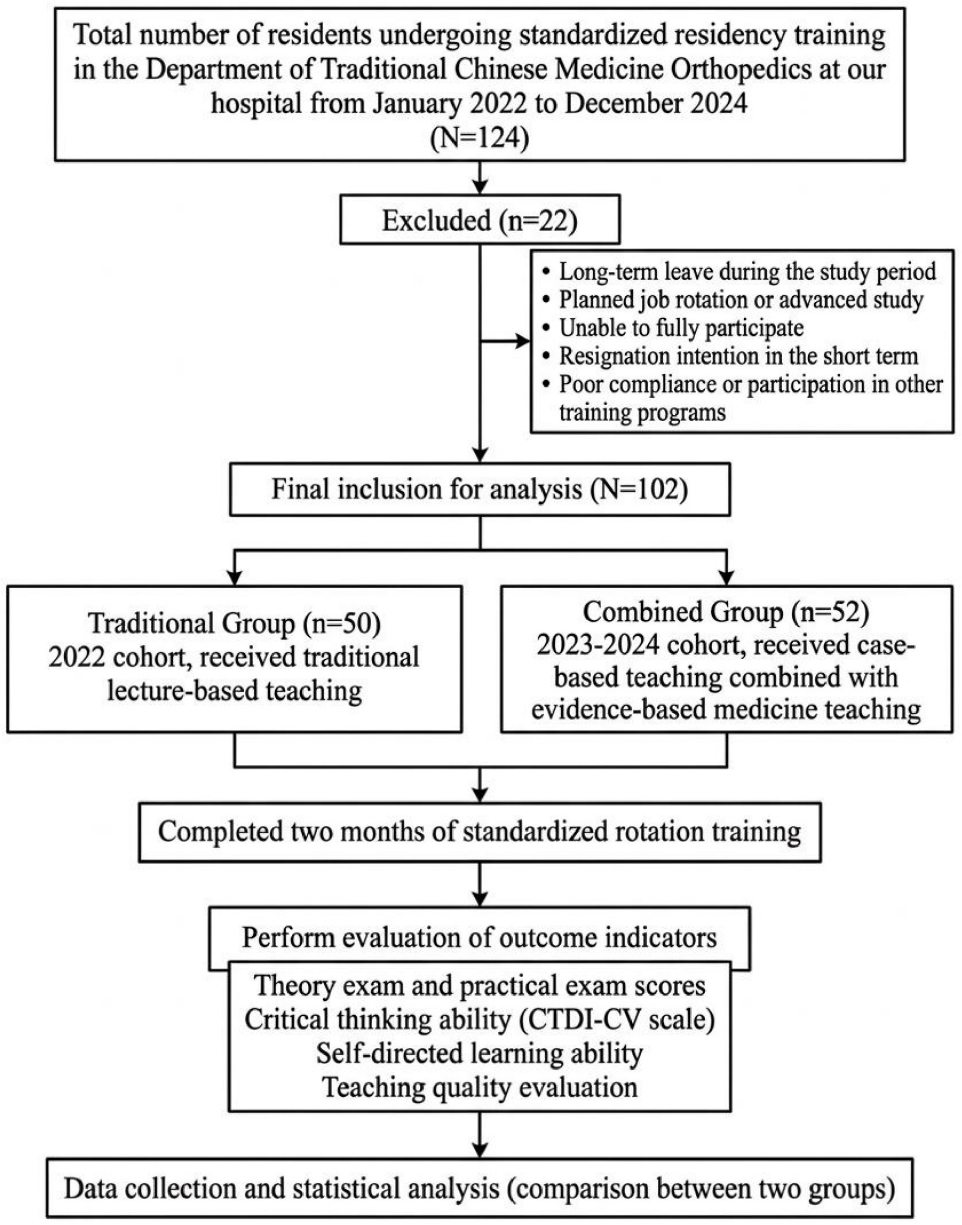
Study flow diagram illustrating the participant selection process and grouping. TCM, traditional Chinese medicine; CBT, case-based teaching; EBM, evidence-based medicine.

This retrospective study included 102 orthopedic residents undergoing residency training in the Department of Traditional Chinese Orthopedics at our hospital from January 2022 to December 2024. Participants were assigned to teaching groups based on the standard training program in effect at the time of their enrollment; thus, allocation was non-random. The cohort was divided into a traditional group (*n* = 50, traditional teaching method, enrolled from January to December 2022) and a combined group (*n* = 52, case-based teaching method combined with evidence-based medicine-based teaching, enrolled from January 2023 to December 2024) according to the sequential implementation of different teaching programs. We acknowledge that this quasi-experimental design, inherent to retrospective educational studies, introduces potential selection bias. We have endeavored to address this by comparing baseline characteristics (Table 1) and discussing its implications in the limitations section. All data were obtained from the hospital management information system. A flowchart summarizing the study design and participant flow is presented in Figure 1.

**TABLE 1:** Comparison of baseline data between the two groups of traditional Chinese medicine orthopedic residents.

Index		Traditional group (*n* = 50)	Joint group (*n* = 52)	χ ^2^	*P*
Sex (n)	Male	35	38	0.119	0.731
	Female	15	14		
Age (years)		25.76 ± 2.25	25.84 ± 2.30	0.177	0.859
Origin of student (n)	Country	10	15	1.078	0.299
	City	40	37		
Whether the child is an only child (n)	Yes	45	45	0.294	0.588
	No	5	7		
Education background (n)	Regular college course	28	34	0.942	0.332
	Bachelor or above degree	22	18		
Reasons for studying medicine (n)	Personal interest	26	29	0.146	0.703
	Adopt sb.’s good points and avoid his shortcomings	24	23		
Do you have any relatives or friends engaged in the medical field (n)	Yes	12	10	0.343	0.558
	No	38	42		
The title of the instructor (n)	Associate senior and above	37	36	0.285	0.593
	Intermediate	13	16		

### 2.3 Inclusion criteria

Inclusion criteria: (1) All participants are residents in the Department of Traditional Chinese Orthopedics at this hospital; (2) Age ≥ 20 years; (3) Hold at least a bachelor’s degree or higher; (4) All teaching is conducted by the same training team; (5) Teaching staff have ≥ 3 years of teaching experience and hold the title of associate senior or above, with the educational title of senior lecturer or above.

Exclusion criteria: (1) Participants with plans to change positions or pursue further education during the study period; (2) Participants unable to fully participate in the study due to personal or unforeseeable circumstances; (3) Participants with intentions to resign in the short term; (4) Participants on long-term leave during the study period; (5) Participants who have undergone other training programs or demonstrate poor compliance.

### 2.4 Teaching methods

The traditional group received a conventional, lecture-based, and apprenticeship-style teaching program, structured as follows: (1) Orientation and Mentorship: Upon entry, residents received a comprehensive orientation from the department chief covering departmental protocols, equipment, and workflow. Each resident was assigned a supervising attending physician (mentor) with the title of associate senior or above. (2) Clinical Observation and Apprenticeship: Residents primarily learned through observation and passive instruction. Mentors performed TCM clinical procedures (e.g., TCM diagnostics, manual therapies, acupuncture) on patients while providing concurrent verbal explanations. Residents’ hands-on opportunities were limited and typically followed a “see one, do one” model under direct supervision only after observing multiple times. (3) Structured Theoretical Input: In addition to bedside teaching, residents attended bi-weekly, 90-min didactic lectures covering core TCM orthopedic theory, common disease patterns (e.g., lumbar disc herniation, cervical spondylosis from a TCM perspective), and principles of TCM treatment modalities. These sessions were delivered by senior faculty using PowerPoint presentations and followed a fixed curriculum without interactive case discussions. (4) Skills Training: A 1-week intensive skills workshop focused on demonstrations of core TCM orthopedic techniques, such as Tui Na (therapeutic massage), acupuncture point localization, and fracture immobilization using traditional methods. Practice was conducted on models or under close supervision on patients. (5) Assessment: After the 2-month rotation, residents’ knowledge was assessed via a 100-point, closed-book written examination covering lecture and textbook material. Practical skills were evaluated through direct observation of procedural skills on standardized patients or simulations, assessing technique and sequence accuracy, but with minimal emphasis on clinical reasoning or evidence justification. The pedagogical approach was predominantly instructor-led, knowledge-transmission focused, with limited structured opportunity for active problem-solving, critical literature engagement, or self-directed inquiry.

The joint group adopts a case-based teaching method combined with evidence-based medicine-based instruction, as follows: (1) Before the course begins, review the relevant content of evidence-based medicine research, with a focus on how to conduct literature searches and evaluations. During the problem-building phase, instructors first present clinically controversial issues, such as how to conduct traditional Chinese medicine diagnostics and treatments for orthopedic patients, then guide students to summarize clinical problems and distill them into answerable clinical questions, thereby enabling faster retrieval of research evidence. (2) The teaching approach was trainee-centered and problem-oriented. Instructors guided participants to collect evidence using EBM methodologies, followed by critical appraisal of the retrieved literature for validity, applicability, and clinical relevance. Using academic databases (e.g., Wanfang, CNKI, VIP, PubMed), instructors curated typical TCM orthopedic clinical cases as the focus for literature searches. Participants were tasked with retrieving relevant domestic and international studies, clinical guidelines, and expert consensus documents. Multiple research findings were synthesized, and reflective questions were posed to the trainees. Key content was delivered via multimedia presentations (e.g., PowerPoint, instructional videos). (3) Prior to training, participants are required to preview the lecture content in advance. The key training content is communicated to participants, and case materials are provided to them before the training. They are instructed to independently conduct relevant literature searches. After the lecture, related questions and learning objectives are assigned to encourage and guide participants in exploration, thereby reinforcing their emotional goals. (4) Strengthen training in traditional Chinese orthopedic skills, including theoretical knowledge of specialized orthopedic diseases, traditional Chinese rehabilitation, acupuncture, and physical therapy, and reinforce the diagnostic and treatment approach of traditional Chinese medicine. (5) Supervising instructors organize trainees to participate in TCM clinical diagnosis and treatment, including observation, auscultation, inquiry, and pulse diagnosis, syndrome differentiation and classification, and the formulation of TCM treatment plans. Supervising instructors observe the trainees, affirm their correct approaches, demonstrate and correct any errors in real time, record the trainees’ performance, and enhance their ability to identify issues and develop clinical reasoning skills. (5) Instructors summarize trainees’ performance throughout the training and practical exercises, organize discussion sessions (without participating themselves), and conduct surveys via questionnaires. Through self-assessment, peer evaluation, and instructor feedback, they assess trainees’ perceived mastery of training content, assess how well students have mastered the knowledge according to the instructors’ and others’ evaluations, summarize issues encountered during the training period, and evaluate students’ acceptance of teaching methods. Based on the evaluation results, adjust the training methods and content accordingly, optimize the training process, and enhance training effectiveness. (6) Finally, during the final discussion and summary session, instructors facilitated an analysis of the evidence presented by the students, integrating the best available evidence with clinical expertise and patient preferences to formulate reasoned clinical decisions.

Intervention fidelity and quality control: To ensure consistency in the delivery of the combined CBT-EBM intervention across the study period, all instructors participated in a standardized training workshop prior to implementation. A detailed teaching manual outlining the curriculum, case protocols, EBM steps, and discussion guides was provided and adhered to. Regular faculty meetings were held to discuss any challenges and maintain consistency. The lead educational supervisor periodically observed teaching sessions to monitor adherence to the protocol. Furthermore, the same core teaching team delivered the program to all participants in the combined group.

The residency training in our department is organized as sequential, 2-month rotational blocks for each cohort of residents. Over the 3-year study period (2022–2024), multiple cohorts underwent training under either the traditional (2022 cohorts) or combined (2023–2024 cohorts) program. All residents within a given cohort received the same teaching method as per the program in effect during their rotation period. Data from all eligible residents across these cohorts were aggregated for analysis by teaching method group.

### 2.5 General data collection

General data for the two groups of students were collected through the hospital management information system, primarily including gender (male/female), age, place of origin (rural/urban), whether they were only children (yes/no), educational background (bachelor’s degree/above), reasons for studying medicine (personal interest/playing to one’s strengths), whether they had relatives or friends working in the medical field (yes/no), and the title of their mentor (associate senior or above/intermediate).

### 2.6 Assessment of examination results

The theoretical and practical examination results of the two groups of traditional Chinese medicine orthopedic residents before and after the training were collected to evaluate the teaching effectiveness. (1) Theoretical results: The two groups of residents were tested on their mastery of theoretical knowledge of common traditional Chinese medicine orthopedic diseases in a closed-book format, with a full score of 100 points. Scores of 85–100 were considered excellent, 60–84 were considered good, and < 60 were considered poor. (2) Practical Assessment: Practical assessments were conducted using standardized patients and scenario simulations, covering two areas: TCM-specific diagnostic and therapeutic techniques and doctor-patient communication. Each area was scored on a scale of 1–50 points, with a total of 100 points. Scores of 85–100 were considered excellent, 60–84 were considered good, and < 60 were considered poor. To minimize bias, outcome assessors (senior faculty not involved in the direct teaching of the participants being assessed) were blinded to the group assignment of the residents during both theoretical examination grading and practical skill evaluations.

### 2.7 Critical thinking skills

Two groups of traditional Chinese medicine orthopedic residents were assessed for their critical thinking skills before and after training using the Chinese version of the Critical Thinking Disposition Inventory (CTDI-CV) developed by Yeh (11). The scale covers seven areas: Analytical thinking, systematic thinking, open-mindedness, curiosity, self-confidence, pursuit of truth, and cognitive maturity, with a total of 70 items. Each item is scored on a scale of 1–6. with a total score ranging from 70 to 420 points. Higher scores indicate higher critical thinking ability. In the present study, the Cronbach’s α coefficient was 0.878, indicating good internal consistency.

### 2.8 Self-directed practice ability

The autonomous practice abilities of the two groups of traditional Chinese medicine orthopedic residents were assessed before and after training using the Self-directed Learning Ability Scale for Medical Students, which has been validated in previous medical education research in China (12). The scale includes six items: self-expression ability, self-learning ability, autonomous interest cultivation, team collaboration ability, innovative adaptability, and clinical diagnostic and therapeutic ability. Each item is scored on a scale of 0–10, with a maximum score of 60. A higher score indicates higher autonomous practice ability. In our sample, the Cronbach’s alpha coefficient was 0.895.

### 2.9 Teaching quality

The teaching quality of two groups of traditional Chinese medicine orthopedic residents before and after training was collected, and a locally developed Teaching Quality Evaluation Scale, which has been used in prior educational studies at our institution and demonstrated acceptable reliability (13), was used to assess the two groups. The scale covered five aspects: learning interest, learning ability, learning attitude, learning thinking, and operational level. Each aspect was scored out of 10 points, with a total teaching quality score of 50 points. The higher the score, the higher the teaching quality. In this study, the Cronbach’s α coefficient was 0.850. The teaching quality questionnaires were administered anonymously and analyzed by researchers not involved in the teaching intervention, ensuring that the analysis was conducted without knowledge of group identity.

### 2.10 Statistical methods

Data were analyzed using SPSS 25.0 statistical software. Count data were expressed as counts (percentages), and χ^2^ Inspection; measurement data that conforms to a normal distribution is expressed as (x̄ ± s), and a *t*-test is used. Given the exploratory nature of this study and that the primary outcomes (exam scores, critical thinking, etc.) represent distinct educational constructs, we did not adjust the alpha level for multiple comparisons, recognizing that this increases the risk of Type I error. However, to aid interpretation, we report effect sizes (Cohen’s d for continuous variables) alongside *P*-values to indicate the magnitude of observed differences. All participants completed the assessments; thus, there were no missing data to handle. A *P*-value of < 0.05 is considered statistically significant.

## 3 Results

### 3.1 Comparison of baseline data between the two groups of traditional Chinese medicine orthopedic residents

There were no statistically significant differences between the two groups in terms of gender, age, place of origin, whether they were only children, educational background, reasons for studying medicine, whether they had relatives or friends working in the medical field, or the professional titles of their mentors (*P* > 0.05), as shown in Table 1.

### 3.2 Comparison of assessment scores between two groups of traditional Chinese orthopedic residency trainees

Theoretical and practical assessment scores serve as direct indicators of teaching effectiveness; higher scores indicate more effective teaching outcomes. The proportion of residents achieving an “Excellent” grade was significantly higher in the combined group compared to the traditional group for both theoretical (67.3% vs. 40.0%) and practical (63.5% vs. 44.0%) assessments. The categorical distributions are detailed in Table 2. The cut-points for “Excellent” (85–100), “Good” (60–84), and “Poor” (< 60) are standardized grading thresholds commonly used in our institution’s assessment system to categorize performance levels.

**TABLE 2:** Comparison of examination results between two groups of traditional Chinese medicine orthopedic residents.

Index	Traditional group (*n* = 50)	Joint group (*n* = 52)	χ ^2^	*P*
Theoretical assessment score				
Excellent	20	35	10.103	0.006
Good	20	15		
Good	10	2		
Practical assessment results				
Excellent	22	33	8.591	0.014
Good	19	18		
Good	9	1		

### 3.3 Comparison of critical thinking ability scores before and after teaching between two groups of traditional Chinese medicine orthopedic residents

Critical thinking ability refers to the ability to evaluate and analyze information, and it is an important means of cultivating students’ independent thinking and judgment. The higher the critical thinking ability score, the stronger the critical thinking ability. Before teaching, there were no statistically significant differences (*P* > 0.05) between the two groups in terms of critical thinking ability scores and total scores for analytical thinking, systematic thinking, open-mindedness, curiosity, self-confidence, pursuit of truth, and cognitive maturity, as shown in Table 3.

**TABLE 3:** Comparison of critical thinking ability scores before teaching between the two groups of traditional Chinese medicine orthopedic residents.

Index	Traditional group (*n* = 50)	Joint group (*n* = 52)	*t*	*P*
Analytical score	34.80 ± 3.52	35.14 ± 3.71	0.474	0.636
Seeking truth score	35.32 ± 3.81	35.59 ± 3.70	0.363	0.717
Systematic score	35.81 ± 3.67	35.54 ± 3.82	0.364	0.717
Curiosity score	37.66 ± 4.07	36.92 ± 3.87	0.941	0.349
Self-confidence score	36.28 ± 3.86	36.35 ± 3.73	0.093	0.926
Openness of thought score	37.10 ± 3.92	36.93 ± 4.06	0.215	0.830
Cognitive maturity score	34.58 ± 3.56	35.12 ± 3.64	0.757	0.451
Total score	252.35 ± 24.20	251.86 ± 25.38	0.100	0.921

**FIGURE 2:**
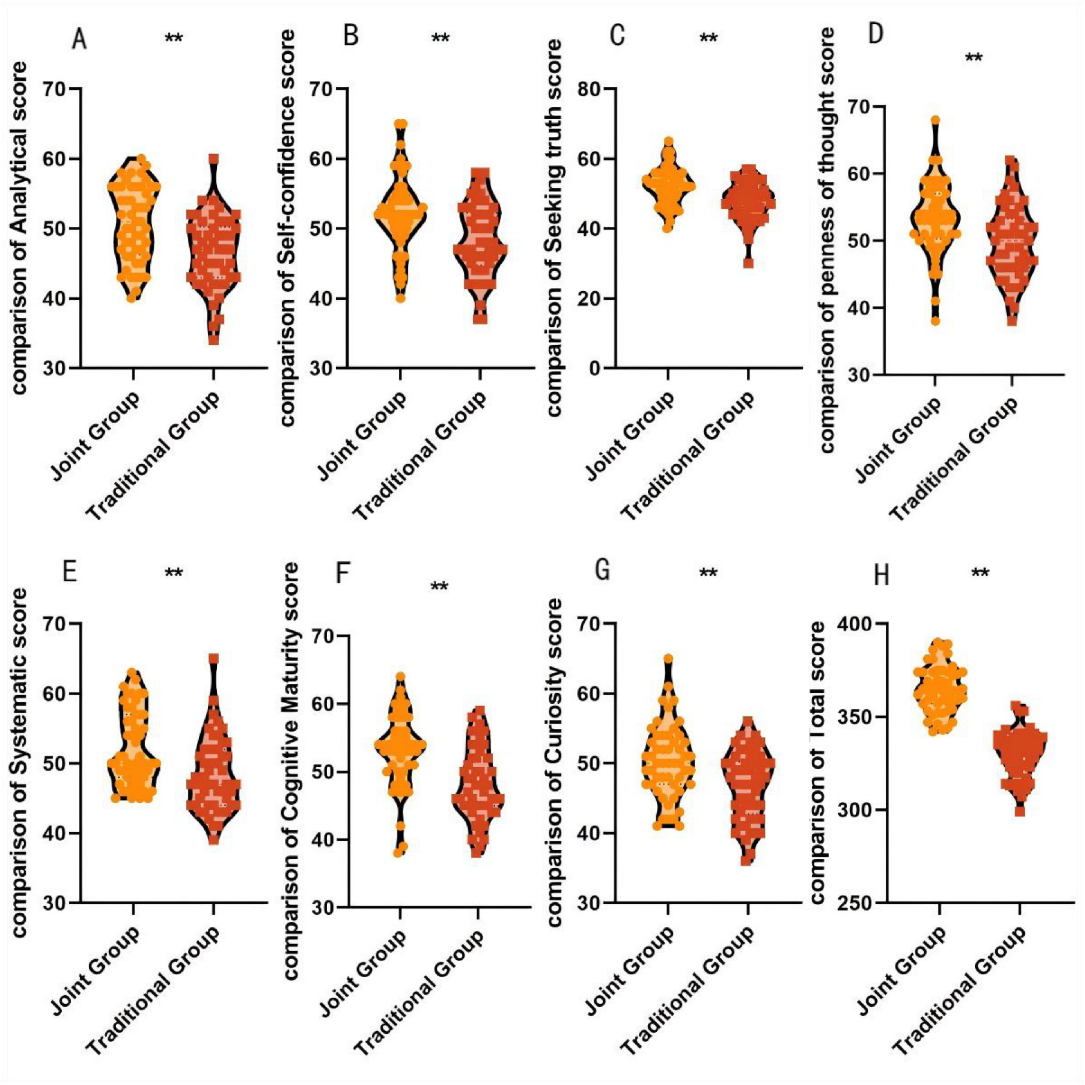
Comparison of critical thinking ability scores between the two groups after teaching. **(A)** Analytical score. **(B)** Self-confidence score. **(C)** Seeking truth score. **(D)** Penness of thought score. **(E)** Systematic score. **(F)** Cognitive maturity score. **(G)** Curiosity score. **(H)** Total score. **P < 0.01.

After the teaching session, the joint group scored 50.69 ± 5.31 vs. 46.54 ± 5.14 for analytical thinking, 52.23 ± 5.29 vs. 47.79 ± 5.42 for truth-seeking, 52.14 ± 5.31 vs. 48.25 ± 5.23 for systematic thinking, curiosity (50.28 ± 5.20 vs. 46.85 ± 4.91), self-confidence (52.05 ± 5.36 vs. 48.25 ± 5.23), open-mindedness (53.23 ± 5.29 vs. 49.79 ± 5.42), and cognitive maturity (53.12 ± 5.34 vs. 48.15 ± 5.26) scores in critical thinking ability and total scores (364.85 ± 12.30 vs. 329.78 ± 12.25) were significantly higher than those of the traditional group, with statistically significant differences (*P* < 0.05), This indicates that the combination of case-based teaching and evidence-based medicine-based teaching in traditional Chinese orthopedic residency training can enhance students’ critical thinking abilities, as shown in Figure 2.

### 3.4 Comparison of self-practice ability scores before and after teaching between two groups of traditional Chinese medicine orthopedic residents

Self-practice ability is an essential key competence in healthcare activities. Higher self-practice ability scores indicate stronger self-practice ability. Before the training, there were no statistically significant differences (*P* > 0.05) in the self-practice ability scores between the two groups in terms of self-expression ability, self-learning ability, self-directed interest development, teamwork ability, innovative adaptability, and clinical diagnostic and therapeutic ability, as shown in Table 4.

**TABLE 4:** Comparison of self-assessment scores for practical skills before and after training between two groups of traditional Chinese medicine orthopedic residents.

Index	Traditional group (*n* = 50)	Joint group (*n* = 52)	*t*	*P*
Self-expression ability score	5.23 ± 1.22	5.36 ± 1.30	0.520	0.604
Self-study ability score	5.22 ± 1.41	5.30 ± 1.38	0.290	0.773
Self-cultivated interest score	5.06 ± 1.40	5.17 ± 1.32	0.408	0.684
Team collaboration ability score	5.24 ± 1.41	5.39 ± 1.35	0.549	0.584
Innovation and adaptability score	5.26 ± 1.27	5.34 ± 1.40	0.302	0.763
Clinical diagnosis and treatment ability score	5.48 ± 1.42	5.56 ± 1.50	0.276	0.783

**FIGURE 3:**
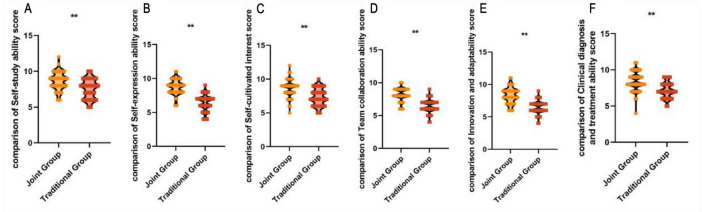
Comparison of self-practice ability scores between the two groups after teaching. **(A)** Self-study ability score. **(B)** Self-expression ability score. **(C)** Self-cultivated interest score. **(D)** Team collaboration ability score. **(E)** Innovation and adaptability score. **(F)** Clinical diagnosis and treatment ability score. **P < 0.01.

After the instruction, the joint group’s self-expression ability (8.70 ± 1.14 vs. 6.40 ± 1.20) score, self-learning ability (8.64 ± 1.28 vs. 7.56 ± 1.40) score, and ability to cultivate interests independently (8.60 ± 1.32 vs. 7.35 ± 1.28) score, team collaboration ability (8.15 ± 1.05 vs. 6.45 ± 1.02), innovative adaptability (8.26 ± 1.25 vs. 7.02 ± 1.22), and clinical diagnostic and therapeutic ability (8.37 ± 1.35 vs. 7.20 ± 1.10) were all higher than those of the traditional group, with statistically significant differences (*P* < 0.05). This indicates that the combination of case-based teaching and evidence-based medicine-based teaching in traditional Chinese orthopedic residency training can enhance students’ self-practice abilities, as shown in Figure 3.

### 3.5 Comparison of teaching quality scores between two groups of traditional Chinese medicine orthopedic residents

**FIGURE 4:**
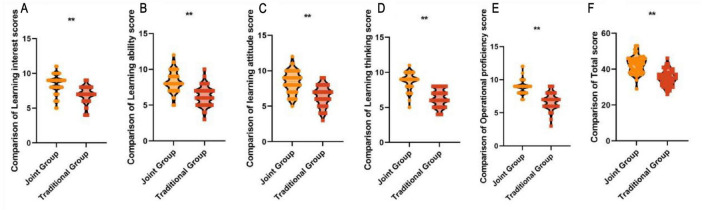
Comparison of teaching quality scores between the two groups. **(A)** Learning interest scores. **(B)** Learning ability score. **(C)** Learning attitude score. **(D)** Learning thinking score. **(E)** Operational proficiency score. **(F)** Total score. **P < 0.01.

Teaching quality scores are a method of comprehensively evaluating the effectiveness, efficiency, and benefits of teaching activities. Higher scores indicate better teaching quality. The joint group scored 8.45 ± 1.38 vs. 6.91 ± 1.20 for learning interest, 8.30 ± 1.45 vs. 6.28 ± 1.35 for learning ability, 8.40 ± 1.52 vs. 6.42 ± 1.48 for learning attitude, learning thinking (8.76 ± 1.17 vs. 6.30 ± 1.25), and operational skills (8.89 ± 1.02 vs. 6.79 ± 1.20) were all higher than those of the traditional group, with statistically significant differences (*P* < 0.05). This indicates that the combination of case-based teaching and evidence-based medicine-based teaching in traditional Chinese orthopedic residency training can improve teaching quality, as shown in Figure 4.

## 4 Discussion

Resident physician standardized training is the final stage of medical student education, and the effectiveness of teaching is closely related to the development of residents’ comprehensive competencies. Traditional Chinese medicine orthopedics involves a wide range of diseases, complex knowledge points, and intricate procedural content, making clinical teaching particularly challenging (14). Traditional teaching methods involve instructors imparting knowledge and skills related to orthopedics, which can achieve some educational outcomes. However, residents are primarily passive learners with low motivation, limited hands-on opportunities, and an inability to integrate theory with practice, leading to gaps in knowledge that can impair future clinical performance and result in suboptimal teaching outcomes (15, 16). Therefore, adopting more reasonable and effective teaching methods is of great significance for improving the effectiveness of TCM orthopedic residency training. This study observed that the combined group performed better than the traditional group in both theoretical and practical assessments. Post-teaching scores for critical thinking ability, self-practice ability, and teaching quality were all higher in the combined group than in the traditional group. These findings suggest that the case-based teaching method combined with evidence-based medicine-based teaching may be more effective for TCM orthopedic residency training, as it promotes students’ critical thinking ability, self-learning ability, and teaching quality.

This study demonstrated that the combined group achieved superior theoretical and practical examination scores compared to the traditional group, suggesting that the integration of case-based teaching methods with evidence-based medicine-based instruction in traditional Chinese orthopedic residency training can enhance students’ theoretical and practical examination performance. Guo et al. (17) found that case-based teaching enables medical students to better integrate theory into practice, thereby enhancing clinical teaching outcomes, which aligns with the conclusions of this study. Ali et al. (18) noted that traditional teaching methods often focus on one-way knowledge transmission, leaving residents passively receiving information and struggling to deeply understand and flexibly apply the complex TCM orthopedic knowledge system. In contrast, the integrated teaching model introduces real or typical clinical cases, placing abstract theoretical knowledge within specific contexts to stimulate residents’ learning interest and curiosity. Additionally, it incorporates evidence-based medicine teaching principles, guiding residents to actively search for, critically evaluate, and apply the best evidence around clinical issues presented in the cases (19). This process forces residents to actively integrate knowledge, connect scattered knowledge points to solve practical problems, not only deepening their understanding and memory of theoretical knowledge but also significantly improving their performance in practical assessments such as standardized patients or scenario simulations, achieving a close integration of theory and practice, and laying a more solid foundation for their future independent clinical work (20).

Our findings are consistent with a significant enhancement in critical thinking skills among residents exposed to the combined CBT-EBM approach, as reflected in higher post-training scores across all subdomains (analytical thinking, systematic thinking, open-mindedness, curiosity, self-confidence, truth-seeking, and cognitive maturity) compared to the traditional group. Critical thinking is indispensable for clinical physicians to navigate complex patient presentations and make evidence-informed decisions (21). Traditional didactic teaching often provides limited opportunity for deep interrogation of clinical problems (22). In contrast, the CBT component of our model presented residents with multifaceted, realistic cases, necessitating active deconstruction of clinical scenarios and identification of core issues. Concurrently, the embedded EBM process-entailing question formulation, systematic evidence retrieval, and critical appraisal-constitutes a rigorous exercise in evaluative thinking (23, 24). Residents were compelled to assess study quality, recognize potential biases, and weigh evidence within a specific clinical context, thereby strengthening their analytical and systematic thinking abilities. This repeated, structured practice in evidence evaluation likely contributed to the observed improvements in cognitive maturity and truth-seeking, skills paramount for TCM orthopedic practitioners when managing complex, variable musculoskeletal conditions (25).

This study used the Self-Directed Learning Ability Assessment Scale to evaluate the self-directed learning abilities of residents. The results showed that the self-directed learning ability scores of the combined group were significantly higher than those of the traditional group after the combined teaching approach. This finding indicates that the combination of case-based teaching and evidence-based medicine-based teaching can effectively stimulate and enhance residents’ self-directed learning abilities. The combined teaching model avoids the issues of the traditional teaching model, which is dominated by the residents’ learning direction and content, with the pace controlled by the teacher, resulting in a strong sense of passivity. Instead, it adopts a problem-oriented, learner-centered approach. After the teacher assigns cases and questions, residents must actively prepare in advance, independently search for information, analyze evidence independently or collaboratively, prepare for discussions, participate in group discussions, and continuously reflect on and adjust their learning strategies under the teacher’s guidance (26). Evidence-based medicine-based teaching emphasizes the process of “seeking answers to questions,” which is inherently an efficient self-directed learning model (27). This teaching model significantly enhances residents’ sense of responsibility for learning, intrinsic motivation, and ability to independently solve problems, laying a critical foundation for them to achieve lifelong learning, continuously update knowledge and skills, and adapt to the rapid development of medicine (19).

The superior teaching quality scores reported by the combined group align with the improvements in learning outcomes and competencies, indicating that the integrated approach was perceived as more effective and engaging. CBT likely heightened learning interest and relevance through contextualized case immersion, thereby positively influencing learning attitudes. EBM contributed by structuring the problem-solving process, thereby optimizing learning thinking and fostering a systematic approach to clinical uncertainty (28). The combined approach does more than transmit knowledge. It also helps cultivate meta-cognitive skills for acquiring and applying knowledge. Furthermore, intensive skill training coupled with evidence-based practice directly targets the enhancement of operational proficiency. Notably, the instructor’s role evolved from a knowledge disseminator to a facilitator and coach, promoting mutual growth through guided discussion, feedback, and iterative process optimization (29). Consequently, the combined model fosters a more dynamic, interactive, and competency-oriented learning environment, which is reflected in the overall higher appraisal of teaching quality.

### 4.1 Limitations

Several important limitations must be considered when interpreting our results. First, the quasi-experimental, retrospective design with non-random group allocation limits causal inference and raises the possibility of selection bias, despite comparable baseline demographics. Second, although we implemented blinding of outcome assessors where feasible, the participants and instructors were not blinded to the intervention, which could influence subjective perceptions (e.g., teaching quality). Third, regarding generalizability (external validity), this study was conducted at a single, large tertiary hospital in China. While this setting provides a controlled environment for initial evaluation, the findings may have limited generalizability to other TCM orthopedic training programs with different institutional resources, faculty-student ratios, cultural contexts, or levels of technological support. The specific traditional teaching method used as a comparator may also vary across institutions. Fourth, the use of multiple outcome measures without statistical adjustment for multiple comparisons increases the risk of Type I error, though we have reported effect sizes to aid interpretation. Fifth, the lack of long-term follow-up means we cannot assess the durability of the observed improvements or their impact on future clinical performance. Finally, while we describe quality control measures, unmeasured variations in teaching delivery over time could have influenced the results.

Therefore, the results should be viewed as preliminary evidence supporting the integrated CBT-EBM approach. To establish its efficacy more robustly and enhance generalizability, future research should prioritize multi-center, prospective, randomized controlled trials with larger sample sizes and long-term follow-up to confirm efficacy, assess cost-effectiveness, and evaluate impact on ultimate clinical outcomes. Additionally, qualitative exploration of learner and faculty experiences could provide valuable complementary insights.

## 5 Conclusion

In this single-center, quasi-experimental study, the combination of case-based teaching and evidence-based medicine-based teaching in traditional Chinese orthopedic residency training was associated with improved teaching effectiveness, as well as higher scores in students’ critical thinking skills, self-directed learning abilities, and teaching quality evaluations compared to traditional lecture-based teaching. While these findings are encouraging and address an important pedagogical gap, the methodological limitations inherent to the retrospective design necessitate cautious interpretation. This study provides preliminary support for the integrated CBT-EBM approach and highlights the need for more rigorous, prospective investigations to confirm its benefits and facilitate broader implementation. This preliminary evidence suggests a promising approach to addressing the challenges posed by the complexity of traditional Chinese orthopedic knowledge points, the strong practical nature of the field, and the tendency of traditional teaching methods to result in a disconnect between theory and practice.

Future Implementation and Research: For educators considering adopting this model, key practical considerations include: the need for faculty development in EBM and case facilitation skills; time investment for case preparation and guided literature searches; and access to bibliographic databases. Future research should prioritize multi-center, prospective, randomized controlled trials with larger sample sizes and long-term follow-up to confirm efficacy, assess cost-effectiveness, and evaluate impact on ultimate clinical outcomes. Qualitative exploration of learner and faculty experiences could also provide valuable insights for optimization.

## Data Availability

The original contributions presented in this study are included in this article/supplementary material, further inquiries can be directed to the corresponding author.

## References

[1] Hou XS Tan C Ning BL Fu WB Zhao JP. Consolidating the foundation, highlighting the practice and strengthening the training of clinical thinking of acupuncture and moxibustion: the thoughts of compiling the China national standardized training textbook Acupuncture and Moxibustion for residents of traditional Chinese medicine. *Zhongguo Zhen Jiu.* (2022) 42:834–8. 10.13703/j.0255-2930.20210612-0003 35793898

[2] Zeng J Liang S Zhang X Yan R Chen C Wen L et al. Assessment of clinical competency among TCM medical students using standardized patients of traditional Chinese medicine: a 5-year prospective randomized study. *Integr Med Res.* (2022) 11:100804. 10.1016/j.imr.2021.100804 35145853 PMC8819389

[3] Han SY Lee SH Chae H. Developing a best practice framework for clinical competency education in the traditional East-Asian medicine curriculum. *BMC Med Educ.* (2022) 22:352. 10.1186/s12909-022-03398-4 35538517 PMC9088070

[4] Guo R Zeng D Zhao Q Zhang XY Zhang XK Liu YL. How are different traditional Chinese medicine modalities deployed by clinical practitioners in China? Findings from a national survey. *J Integr Med.* (2025) 23:36–45. 10.1016/j.joim.2024.11.004 39613684

[5] Yang H Xiao X Wu X Fu X Du Q Luo Y et al. Virtual standardized patients versus traditional academic training for improving clinical competence among traditional Chinese medicine students: prospective randomized controlled trial. *J Med Internet Res.* (2023) 25:e43763. 10.2196/43763 37728989 PMC10551797

[6] Manas A Ismail PMS Mohan R Madiraju GS Mulla M Mulla M et al. The role of combined problem-based learning (PBL) and case-based learning (CBL) teaching methodologies in dental education. *J Educ Health Promot.* (2024) 13:417. 10.4103/jehp.jehp_1824_23 39703663 PMC11658036

[7] Li F Luo J Zhang H. The application of problem-based learning combined with case-based learning in EEG teaching. *J Med Educ Curric Dev.* (2024) 11:23821205241252277. 10.1177/23821205241252277 38711831 PMC11072060

[8] Xie W Li Y Liu X. Application of problem-based learning and case-based learning in teaching ectopic pregnancy to fifth-year medical students. *BMC Med Educ.* (2024) 24:1346. 10.1186/s12909-024-06327-9 39574098 PMC11583762

[9] Yan X Zhu Y Fang L Ding P Fang S Zhou J et al. Enhancing medical education in respiratory diseases: efficacy of a 3D printing, problem-based, and case-based learning approach. *BMC Med Educ.* (2023) 23:512. 10.1186/s12909-023-04508-6 37461009 PMC10353117

[10] Peng Y Yang L Qi A Zhang L Xiong R Chen G. Simulation-based learning combined with case and problem-based learning in the clinical education of joint surgery. *J Surg Educ.* (2023) 80:892–9. 10.1016/j.jsurg.2023.03.001 37032261

[11] Yeh ML. Assessing the reliability and validity of the Chinese version of the California critical thinking disposition inventory. *Int J Nurs Stud.* (2002) 39:123–32. 10.1016/s0020-7489(01)00019-0 11755443

[12] Lim YS Lyons VT Willey JM. Supporting self-directed learning: development of a faculty evaluation scale. *Teach Learn Med.* (2022) 34:494–503. 10.1080/10401334.2021.1977136 34645314

[13] Wang J Zang S Shan T. Dundee ready education environment measure: psychometric testing with Chinese nursing students. *J Adv Nurs.* (2009) 65:2701–9. 10.1111/j.1365-2648.2009.05154.x 19941551

[14] Chan PP Lee VWY Yam JCS Brelén ME Chu WK Wan KH et al. Flipped classroom case learning vs traditional lecture-based learning in medical school ophthalmology education: a randomized trial. *Acad Med.* (2023) 98:1053–61. 10.1097/ACM.0000000000005238 37067959

[15] Gholami M Changaee F Karami K Shahsavaripour Z Veiskaramian A Birjandi M. Effects of multiepisode case-based learning (CBL) on problem-solving ability and learning motivation of nursing students in an emergency care course. *J Prof Nurs.* (2021) 37:612–9. 10.1016/j.profnurs.2021.02.010 34016321

[16] Yang W Zhang X Chen X Lu J Tian F. Based case based learning and flipped classroom as a means to improve international students’ active learning and critical thinking ability. *BMC Med Educ.* (2024) 24:759. 10.1186/s12909-024-05758-8 39010040 PMC11247815

[17] Guo QY Ke WJ Zeng ZX Liu J Chen M Qiu Q. Application of the combined problem-based learning and case-based learning teaching in urinalysis course of diagnostics. *MedEdPublish.* (2024) 14:43. 10.12688/mep.20266.1

[18] Ali N Dhere TA Bates JE Lorenz JW Janopaul-Naylor JR Schlafstein AJ et al. Integration of radiation oncology into the preclinical curriculum through problem-based learning. *Pract Radiat Oncol*. (2024) 14:e1–8. 10.1016/j.prro.2023.08.013 37802397

[19] Fromke EJ Jordan SG Awan OA. Case-based learning: its importance in medical student education. *Acad Radiol.* (2022) 29:1284–6. 10.1016/j.acra.2021.09.028 35835535

[20] Nishal A Patel JN Balvalli R Yadav PP Jayani P Singh R et al. A comparative study of case-based learning vs. traditional teaching method in pathology in Indian medical graduates. *J Med Educ.* (2022) 21:268–75. 10.5812/jme-127188

[21] Yan J Wen Y Liu X Deng M Ye B Li T et al. The effectiveness of problem-based learning and case-based learning teaching methods in clinical practical teaching in TACE treatment for hepatocellular carcinoma in China: a Bayesian network meta-analysis. *BMC Med Educ.* (2024) 24:1025–35. 10.1186/s12909-024-05615-8 38886707 PMC11184776

[22] Huang M Yang H Guo J Fu X Chen W Li B et al. Faculty standardized patients versus traditional teaching method to improve clinical competence among traditional Chinese medicine students: a prospective randomized controlled trial. *BMC Med Educ.* (2024) 24:793. 10.1186/s12909-024-05779-3 39049066 PMC11267817

[23] Zhou F Yuan T Li Z Mu X Lv Y. The evidence-based practice teaching competence of clinical preceptors at different stages of innovation-decision process: a cross-sectional survey in traditional Chinese medicine hospitals. *Nurse Educ Today.* (2024) 132:106027. 10.1016/j.nedt.2023.106027 37956570

[24] Li Y Ren F Qiu J Li D Wang J Lin S. Effectiveness of a BOPPPS teaching model in standardized training for nephrology resident physicians: a retrospective cohort study. *Br J Hosp Med*. (2024) 85:1–13. 10.12968/hmed.2024.0437 39831479

[25] Lyu X Zhao J Tang R Pan H Chen S. Comparison of traditional face-to-face teaching with synchronous distance education in medical theory courses teaching to medical undergraduates: a case-controlled study in China. *Medicine.* (2024) 103:e40714. 10.1097/MD.0000000000040714 39654185 PMC11630938

[26] Liu CX Ouyang WW Wang XW Chen D Jiang ZL. Comparing hybrid problem-based and lecture learning (PBL + LBL) with LBL pedagogy on clinical curriculum learning for medical students in China: a meta-analysis of randomized controlled trials. *Medicine.* (2020) 99:e19687. 10.1097/MD.0000000000019687 32311943 PMC7220526

[27] Shrestha B Subedi S Paudel S Subedi N Parajuli U. Impact of case based learning on teaching of undergraduate oral pathology course. *J Nepal Health Res Counc.* (2023) 21:238–42. 10.33314/jnhrc.v21i02.4312 38196214

[28] Gasim MS Ibrahim MH Abushama WA Hamed IM Ali IA. Medical students’ perceptions towards implementing case-based learning in the clinical teaching and clerkship training. *BMC Med Educ.* (2024) 24:200. 10.1186/s12909-024-05183-x 38413966 PMC10900817

[29] Hu B Wang L Wu J Zhu L Chen Z. A combination of case-based learning with flipped classroom improved performance of medical students in nephrology bedside teaching. *BMC Med Educ.* (2024) 24:995. 10.1186/s12909-024-05973-3 39266995 PMC11396661

